# A cluster randomised controlled trial, process and economic evaluation of quality improvement collaboratives aligned to a national audit to improve the care for people with diabetes (EQUIPD): study protocol

**DOI:** 10.1186/s13012-023-01293-0

**Published:** 2023-08-31

**Authors:** Michael Sykes, Bethan Copsey, Tracy Finch, David Meads, Amanda Farrin, Jenny McSharry, Naomi Holman, Bob Young, Alex Berry, Kat Ellis, Lauren Moreau, Thomas Willis, Sarah Alderson, Melissa Girling, Elaine O’Halloran, Robbie Foy

**Affiliations:** 1 Northumbria University, Newcastle Upon Tyne, UK; 2 University of Leeds, Leeds, UK; 3 University of Galway, Galway, Ireland; 4 Imperial College London, London, UK; 5 National Diabetes Audit, Leeds, UK; 6 DiabetesUK, London, UK; 7 Expert By Experience, London, UK

**Keywords:** Audit and feedback, Quality Improvement Collaborative, Diabetes, Insulin pump, Randomised controlled trial

## Abstract

**Background:**

People with type 1 diabetes and raised glucose levels are at greater risk of retinopathy, nephropathy, neuropathy, cardiovascular disease, sexual health problems and foot disease. The UK National Institute for Health and Care Excellence (NICE) recommends continuous subcutaneous ‘insulin pump’ therapy for people with type 1 diabetes whose HbA1c is above 69 mmol/mol. Insulin pump use can improve quality of life, cut cardiovascular risk and increase treatment satisfaction. About 90,000 people in England and Wales meet NICE criteria for insulin pumps but do not use one. Insulin pump use also varies markedly by deprivation, ethnicity, sex and location. Increasing insulin pump use is a key improvement priority.

Audit and feedback is a common but variably effective intervention. Limited capabilities of healthcare providers to mount effective responses to feedback from national audits, such as the National Diabetes Audit (NDA), undermines efforts to improve care. We have co-developed a theoretically and empirically informed quality improvement collaborative (QIC) to strengthen local responses to feedback with patients and carers, national audits and healthcare providers. We will evaluate whether the QIC improves the uptake of insulin pumps following NDA feedback.

**Methods:**

We will undertake an efficient cluster randomised trial using routine data. The QIC will be delivered alongside the NDA to specialist diabetes teams in England and Wales. Our primary outcome will be the proportion of people with type 1 diabetes and an HbA1c above 69 mmol/mol who start and continue insulin pump use during the 18-month intervention period. Secondary outcomes will assess change in glucose control and duration of pump use. Subgroup analyses will explore impacts upon inequalities by ethnicity, sex, age and deprivation. A theory-informed process evaluation will explore diabetes specialist teams’ engagement, implementation, fidelity and tailoring through observations, interviews, surveys and documentary analysis. An economic evaluation will micro-cost the QIC, estimate cost-effectiveness of NDA feedback with QIC and estimate the budget impact of NHS-wide QIC roll out.

**Discussion:**

Our study responds to a need for more head-to-head trials of different ways of reinforcing feedback delivery. Our findings will have implications for other large-scale audit and feedback programmes.

**Trial registration:**

ISRCTN82176651 Registered 18 October 2022.

**Supplementary Information:**

The online version contains supplementary material available at 10.1186/s13012-023-01293-0.

Contributions to the literature

- Theory and policy recommend increasing the quality improvement capabilities of audit and feedback recipients.
- We will test a theory-, evidence-, and stakeholder-informed Quality Improvement Collaborative (QIC) to enhance recipients’ capabilities to respond to the National Diabetes Audit.
- We will evaluate the effect of QIC on the use of insulin pumps, equity of uptake, patient outcomes and cost-effectiveness, as well as explore implementation and engagement with the QIC.
- Identifying target practices for recipients of feedback from audits, and how to implement these, offers the opportunity for improvements in care and valuable learning to national audit commissioners and providers, and implementation researchers.

## Background

Over 192,000 people in England have type 1 diabetes, almost half of whom have HbA1c levels above 69 mmol/mol putting them at greater risk of retinopathy, nephropathy, neuropathy, cardiovascular disease, sexual health problems and foot disease [1]. The UK National Institute for Health and Care Excellence (NICE) recommends continuous subcutaneous insulin infusion ('insulin pump') therapy for people with type 1 diabetes whose HbA1c is above 69 mmol/mol despite receiving a high level of care [2].

Insulin pump use can improve quality of life [3], reduce cardiovascular risk [4], and increase treatment satisfaction [5]. Yet the National Diabetes Audit (NDA) demonstrates slow and unequal progress in uptake of insulin pumps; around 90,000 people who meet NICE criteria are not prescribed insulin pumps (NDA) [6]. Pump use varies markedly by locality (2% to 47%; [1]), by deprivation (16.3% most deprived; 23.8% least deprived; [1]) and by ethnicity [7]. The NDA has identified accelerating the uptake and equality of insulin pump use as a key priority in reducing mortality and morbidity [1].

The NDA is one of around 60 national audits in England [8]. Funded by the National Health Service (NHS) in England and Wales, it provides feedback on recommended processes of care (e.g. proportions of people with diabetes having foot checks) and attainment of treatment goals (e.g. blood sugar or blood pressure control) to specialist teams. Feedback from the audit highlights areas for improvement to stimulate change. Whilst audit and feedback has shown modest improvements on care delivery [9], there are considerable opportunities to improve the impact of national audit programmes such as the NDA by, for example, incorporating goals and action plans for change [10]. However, a common challenge amongst those leading national audits is that even well-designed feedback may only have limited impact in the absence of robust local quality improvement arrangements. For example, we found no improvements in care from enhanced feedback reports in two trials embedded within a national audit programme which aimed to reduce inappropriate blood transfusions; a major reason for the absence of any improvement was the lack of effective local responses to feedback [11]. Evidence (e.g. [12, 13]), theory [14] and stakeholder prioritised hypotheses [15] highlight an opportunity to increase the effectiveness of national audits by enhancing the ability of feedback recipients to mount concerted quality improvement efforts.

The Healthcare Quality Improvement Partnership (HQIP), the main commissioner of national audits in England, states that, “health care providers require additional support to make best use of performance feedback data. This is likely to be most effective as part of a coordinated regional or national improvement programme” [12]. Several frameworks propose that significant improvements in care can only be achieved by launching and coordinating quality improvement efforts across all levels of healthcare systems (national, organisational, team, and individual) [16, 17]. Whilst national audit may provide the impetus for change at clinical team and individual levels, there is often insufficient local organisational capability to enable change by, for example, systematically aligning actions to barriers to, and levers for, improvement [14]. Local quality improvement may also be undermined by a lack of motivation to change [14, 16, 18], limited opportunities for improvement actions [16, 19] and poor adaptability to local organisational context [20]. HQIP guidance states “national clinical audits need to be put into the local context to inform action plans addressing areas where quality improvements can be made” (p8; [12]).

We have developed a quality improvement collaborative (QIC) that supports providers to improve by selecting actions tailored to local contexts and generating organisational commitment for change [19]. Intervention development [21] included multi-method co-design to understand current responses to national audits and further co-design of a stakeholder-, theory- and evidence-informed intervention to support recipients.

We have explored the feasibility, acceptability, appropriateness and fidelity of this intervention when delivered with two national audits (NDA and National Audit of Dementia [19]). We recently delivered the intervention to 28 diabetes teams and evaluated feasibility, appropriateness, fidelity and scalability. We found that the behaviour change techniques [22] identified in the manual were delivered by facilitators. There was evidence for fidelity of enactment of target behaviours. Participants reported positive attitudes towards the intervention and that the intervention was appropriate [19]. We consider that the intervention is now ready for a definitive effectiveness evaluation.

We will evaluate the effectiveness of NDA feedback with QIC compared to NDA feedback alone, understand intervention implementation, engagement, fidelity and tailoring of actions, and estimate value for money of NDA feedback with QIC.

## Methods

### Study design

**Fig. 1:**
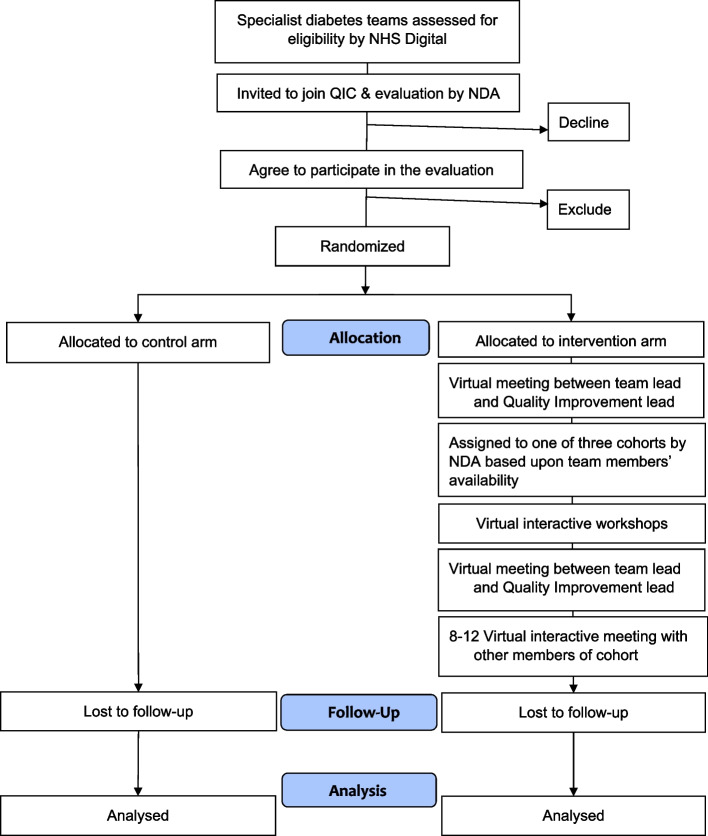
CONSORT flow chart

EQUIPD (Evaluation of Quality Improvement for People with Diabetes) is an efficient cluster randomized controlled trial with parallel process and economic evaluations using routine NDA data. One hundred twenty specialist diabetes teams (clusters) will be allocated on 1:1 basis to either control (NDA feedback alone) or intervention (NDA feedback plus QIC) arms (Fig. 1). Control arm teams will receive the intervention after the study follow-up period, but prior to completion of data analysis.

### Study setting

Specialist diabetes teams from England and Wales providing care in community and in-patient settings. These multidisciplinary teams include diabetologists, endocrinologists, diabetes specialist nurses and dieticians.

### Cluster eligibility

Specialist diabetes teams which accept an invitation to participate in the QIC will be eligible.

### Patient eligibility

Our patient population will be all people identified through the national audit before the intervention period (baseline) with an HbA1c level above 69 mmol/mol and not prescribed insulin for a pump in the previous year.

### Randomisation and blinding

Participating specialist teams will be randomised after agreement to participate and confirmation of eligibility. Specialist diabetes teams (clusters) will be independently randomised on a 1:1 basis to receive the intervention or waitlist only control (receiving the intervention after the trial follow-up period ends), using a computer-generated minimisation programme.

Minimisation factors: Baseline proportion moving onto a pump in the 15 months prior to the intervention period (above or below median); size of target patient population in specialist team (above or below median); previous QIC pilot participation [19] (yes or no).

Participating specialist teams will not be blinded to allocation.

### Intervention

Teams allocated to the intervention arm will receive standard NDA feedback (described in Supplementary materials 1) and the QIC to promote the uptake of insulin pumps.

The QIC will be delivered through two virtual workshops (6 h in total), two virtual outreach sessions (30 min) and virtual facilitated multisite meetings (1 h each). Delivery will be by the NDA Quality Improvement Lead, the DiabetesUK Engagement Lead, the NDA Clinical Lead and clinicians with expertise in improvement and insulin pump use. The intervention will be delivered virtually via Microsoft Teams and Google JamBoard.

We will ask participating teams to attend workshops and ensure a team member attends at least 8 of the 12 multisite meetings. Teams are supported over a 15-month period. Only identified, invited members from participating sites will have access the virtual workshops and meetings. Delivery will be in three parallel cohorts of 20 teams, with each cohort forming a ‘collaborative.’ Learning will be shared between cohorts by the facilitator. Teams will be asked to identify a replacement if any team member leaves. The replacement will be offered a 30-min one-to-one call to support within-team discussions about the intervention.

**Fig. 2:**
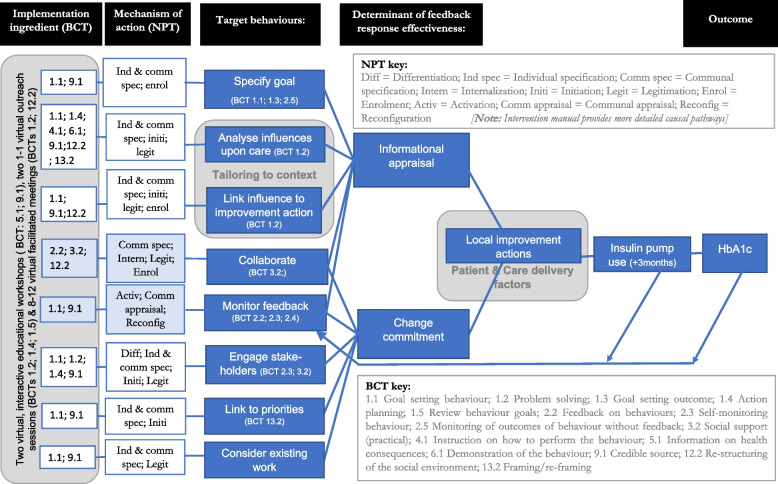
EQUIPD Protocol

The logic model (Fig. 2) outlines the behaviours targeted by the intervention. It also describes the mechanisms that influence their implementation and the behaviour change techniques intended to address these mechanisms.

Intervention content and delivery are described in the TIDieR framework [23] (Appendix) and logic model (Fig. 2) and an intervention manual. In summary, the intervention supports teams to specify a goal; analyse influences upon care; link influence to the improvement action; collaborate; review feedback; engage stakeholders; link the work to priorities; and consider existing work. We anticipate that enactment of these target behaviours will provide the informational appraisal to select effective actions and generate organisational commitment needed to bring about those actions.

### Follow-up

We have chosen an 18-month follow-up period for two reasons. First, it reflects the duration of the QIC, plus 3 months to assess sustainment. Second, it allows for a period for teams engaging with the QIC to consider, plan and initiate changes in clinical practice.

All teams will be given the opportunity to receive the intervention alongside standard NDA feedback. It is not possible to support all teams at once. We will therefore randomly allocate half to receive the intervention, whilst the other half will receive the intervention 18 months later (control group) 3 months after intervention arm delivery is completed. Teams that choose not to be included in the evaluation will receive the intervention alongside the control group.

Allocation concealment is not feasible in this trial. We will maintain a log of those unblinded to allocation. This will include the intervention delivery team and those research team members who need to know allocations to undertake the study. The intervention supports teams to engage local patients to identify potential barriers and actions to improvement, as such patients may not be blinded to involvement in the intervention arm.

### Study procedure

Each cluster will be a clinical team providing specialist care to adults with diabetes, as identified by NHS Digital. Specialist teams will be invited both to be part of the QIC and to participate in the evaluation by the NDA.

We plan to recruit 60 specialist teams per arm, each including an average of 600 patients with type I diabetes with HbA1c > 69 mmol/mol not using a pump within the previous year.

Sample size calculation and the assumed intra-class correlation co-efficient (ICC) is described in Supplementary materials 1.

### Recruitment

Clinical leads will give consent on behalf of their specialist diabetes teams. As part of usual care, the NDA will email all specialist teams to invite participation in the QIC. The invitation will describe the evaluation and ask whether they, as a team, wish to be included in the evaluation. Further recruitment and withdrawal procedures are described in the Supplementary materials 1.

### Primary and secondary outcomes

The primary outcome is the proportion of adults with type 1 diabetes and raised glucose levels (HbA1c above 69 mmol/mol) starting and continuing to use insulin pumps for at least 3 months within an 18-month follow-up period.

Our secondary outcomes are:

- Change in blood glucose levels as measured by HbA1c in people with type 1 diabetes and raised glucose levels between the latest measurement in the 12 months preceding the start of the intervention and the latest measurements recorded during the study period.
- Any record of insulin pump prescribing, including for periods shorter than 3 months.
- Insulin pump use sustained over at least 6 months.

### Data collection

Trial outcomes and demographic data will be assessed using routinely collected individual patient data already extracted for the NDA. A statistician embedded within the NDA team at NHS England (previously NHS Digital) will analyse individual patient-level data with supervision from a researcher experienced in using NDA data and by the senior trial statistician. Supplementary materials 1 details data extraction.

From the audit data, we will define a closed cohort of participants who have an HbA1c level above 69 mmol/mol and were not prescribed insulin for a pump in the previous year.

Data will be summarised on the number of specialist teams invited to participate, agreed to participate, and are randomised. For randomised clusters, based on data from the NDA, we will summarise the baseline proportion of patients moving onto a pump in the 15 months prior to the intervention period and the size of target patient population in specialist team. We will also summarise the number and proportion of teams who previously participated in the QIC pilot. The target patient population will be people with an HbA1c level above 69 mmol/mol and not prescribed insulin for a pump in the previous year. We will summarise the age, sex, ethnicity and deprivation of patients. We will extract data from the 15 months prior to randomisation and the 18 months following randomisation.

For the intervention sessions, data will be collected on the timing, mode of delivery and duration of each session. Data will also be collected on the attendance at each session (by team and job title). Intervention content will be assessed as described in the fidelity assessment. Participant engagement will be assessed as described in the process evaluation.

All patient-level data will come from the NDA.

### Data monitoring

The NDA validates, monitors and reports the quality of patient outcome and demographic data [24]. Data regarding randomization will be monitored for quality and completeness by the clinical trials unit, using established verification, validation and checking processes.

### Data analysis

We will use all available data from all randomised specialist teams, according to a detailed pre-specified plan finalised and agreed by the research team before any analyses are undertaken. We will conduct all analyses on the intent-to-treat (ITT) population, including all specialist teams and patients in the group they are randomised to regardless of intervention adherence.

We will conduct a single final analysis after the end of the follow-up period, when fully cleaned data are available from the NDA. Blinded interim reports will be presented to the Project Steering Committee (PSC) containing descriptive information on site recruitment and intervention adherence.

### Summary of intervention data

Quantitative summaries of intervention delivery will be presented, including timing and mode of delivery of sessions, uptake and engagement.

Summary statistics will be presented for baseline data by treatment group using means, standard deviations, medians, minimum, maximum, and quartiles for continuous variables, and counts and percentages for categorical variables. Summaries will be presented at the specialist team level where appropriate.

We will compare characteristics of patients lost to follow-up with those not lost to follow-up to assess for attrition bias.

### Primary outcome analysis

The primary ITT analysis will compare the primary outcome between trial arms, using mixed effects logistic regression, with patients nested within specialist teams, and with specialist teams treated as a random intercept, adjusting for patient-level and team-level covariates (including patient age, sex, ethnicity, deprivation and team-level stratification factors). Estimated mean odds ratios will be reported with 95% confidence intervals, *p* values and intra-cluster correlation coefficients.

### Subgroup analysis

Planned exploratory subgroup analyses will explore potential moderators of primary outcome treatment effect using key baseline factors: age, sex, ethnicity, and deprivation. This will indicate whether the QIC contributes towards reducing inequalities in care. Subgroup analyses are exploratory, providing estimates of the direction and size of any interactions.

### Secondary outcome analysis

For binary secondary outcomes (any insulin pump prescribing, sustained insulin pump prescribing), mixed effects logistic regression will be used, using the same approach as for the primary outcome.

For continuous secondary outcomes (glucose levels measured by HbA1c), we will use mixed effects linear regression and report estimated mean differences with 95% confidence intervals, *p* values and intra-cluster correlation coefficients.

### Missing data

Although we expect the level of missing data to be small, we will investigate patterns of missing data and reasons for missing data. We will compare the proportions of missing data between intervention and control groups.

We will build a multiple imputation model assuming data is missing at random for the primary outcome. A sensitivity analysis will consider a scenario where participants with missing outcome data are assumed not to move onto an insulin pump.

### Process evaluation

Our theory-informed, integrated process evaluation will comprise semi-structured interviews, surveys, documentary analysis and observations, with work package level analysis and synthesis.

Guided by the MRC Framework for developing and evaluating complex interventions, our objectives are to:

- Describe how implementers engage with the QIC intervention overall to support improvement activity and how context influences this work (implementation and engagement).
- Assess fidelity of delivery, receipt and enactment of the QIC intervention (fidelity).
- Describe how teams enact tailoring (tailoring).

### Theoretical approach

The evaluation will draw upon the same theories applied in developing the QIC intervention: Organisational readiness to change theory [17] to describe the target behaviours undertaken by intervention recipients; Normalisation Process Theory (NPT) [25] to explore implementation, and Behaviour Change Techniques (BCTs) [22] to describe delivery (Fig. 2).

The process evaluation will investigate whether hypothesised mechanisms for achieving change in both professional behaviour (what intervention recipients do) and patient-level outcomes (the use of insulin pumps) are evident when the QIC is used in practice, and what wider (and perhaps unanticipated) factors affect these mechanism-outcome relationships.

The logic model outlines the BCTs intended to trigger NPT mechanisms within the QIC intervention and targeted behaviours of specialist diabetes teams. We will draw on this logic model and the fidelity assessment approach of Lorencatto et al. [26] to assess the extent to which intervention components are delivered by intervention facilitators and received by specialist diabetes teams and if targeted behaviours are enacted as intended.

The QIC supports teams to explore influences upon performance, identified using the Theoretical Domains Framework (TDF) [27] and to select improvement actions aligned to these influences. Previously identified influences upon insulin pump use include patient factors (e.g. knowledge and skills), staff factors (motivation; beliefs about acceptability to, and consequences for, patients; beliefs about capacity and capability) and contextual factors (e.g. culture, funding, time). The tailoring evaluation will describe the selected improvement actions using the Expert Recommendations for Implementing Change (ERIC) [28] compilation of implementation strategies, and the template for intervention description and replication (TIDieR) framework. The analysis will be ongoing and iterative and draw upon other approaches from implementation science where relevant.

### Participants

We will include both specialist diabetes teams (typically including diabetologists and nurses) taking part in the study, and staff delivering the QIC intervention.

### Sampling and recruitment

We will sample participants from both intervention and control arms, weighted towards the former and aiming to ensure diversity of teams, service settings and patient population characteristics.

To limit participant burden, we will sample from around half (30) of the intervention teams for interviews, but from all intervention teams for observations and documentary analysis. Documents will include those produced within QIC workshops (for which we will give authors the opportunity to have their data excluded from the analysis) and organisational documents (e.g. local reports with appropriate permissions).

For interviews, we will undertake strategic sampling within the intervention arm, for example, by baseline proportion moving onto a pump in the 15 months prior to the intervention period (above or below median) and number of patients served by the specialist team (above or below median). We will sample from 8 to 12 control arm teams.

### Data collection

We will collect data from both intervention and control (teams assigned to later receipt) arms to explore ‘implementation as usual’ work for the NDA and any potential contamination between study arms. We will use four qualitative methods: around 60–80 theory-informed, semi-structured interviews, 4–8 surveys, analysis of up to around 120 documents and an estimated 55 h or less of observations. Where appropriate, the data will be used for all three process evaluation objectives (process and engagement, fidelity, tailoring) and the WP3 economic evaluation.

We will interview diabetes team members at multiple time points. The interviews will use a topic guide developed from our aforementioned theoretical approaches, including open questions for exploring barriers, facilitators and mechanisms related to NPT and BCTs. Interviews will also include more tailored questioning, developed from prior analysis of other data sources (documents and observation), and serving as prompts for more detailed investigation. An initial round of approximately 20 interviews will include both intervention deliverers and intervention arm participants early during intervention delivery to assess intervention engagement, fidelity, and tailoring. A further 40–50 interviews towards the end of the intervention period will seek more reflective data on intervention engagement and perceptions (including tailoring) and data required to complete fidelity assessment. This latter set of interviews will include 8–12 interviews with control arm participants to focus on team experiences of undertaking quality improvement in relation to the NDA in the absence of intervention, thus providing contextual data about ‘implementation as usual’. Combined with data from intervention participants, this will also provide scope to explore and understand any contamination across trial arms (e.g. if control participants mention access to QIC intervention documentation).

We will collect up to 55 h of observational data with intervention participants only, contributing data on implementation, engagement, fidelity and tailoring. This will capture participant descriptions of both planned and completed improvement actions. The observations will include recorded virtual interactive educational workshops, virtual outreach sessions and multisite facilitated meetings. Around two thirds of these data will be captured within the first 3 months of intervention delivery, allowing later interviews to focus more directly on issues arising from the observations. We will record intervention exercises and monthly virtual facilitated meetings for subsequent structured observational analysis. We will conduct more focused qualitative observations at some sites (identified at interview), to include meetings of implementation teams and meetings with key stakeholders (either in real time or recordings), as available and appropriate.

Around 120 documents for analysis will include materials concerning intervention exercises and activities, including stakeholder maps, logic models, action plans, and summaries of any discussions and meetings available to the research team. We anticipate the majority of these will concern intervention arm participants and provide additional detail for assessing fidelity of delivery, receipt and enactment and understanding tailoring activity. We will seek some documentation for analysis from control arm participants towards the end of the intervention period. These documents will be identified during control participant interviews and requested for inclusion in the study if available. They will likely comprise internal quality assurance reports and will help understand ‘implementation as usual’.

We will use an online survey to collect data from those intervention deliverers who provide brief input into intervention delivery. The survey will be emailed within the first 3 months of intervention delivery.

### Data analysis

Iterative analysis of interview data will use both inductive and deductive approaches, according to standard procedures [29, 30]. For example, the fidelity assessment will deductively seek the presence or absence of the enactment of target behaviours; the evaluation of implementation will inductively explore influences on engagement. Documents will be read in parallel by two researchers who will extract data for analysis according to the different process evaluation objectives. For implementation and engagement, these documents will be used to develop prompts and more detailed questions within the topic guide when interviewing the team members who authored them. For investigation of tailoring processes, documents will prompt questioning about the influences teams identified and how (and why) they linked these to their documented strategies. For fidelity, documentary analysis will focus on enactment of target intervention behaviours (e.g. use of the TDF to identify influences; the development of a list of stakeholders for engagement). We will take a more structured approach for observational data, assessing fidelity through coding and comparing the BCTs in the manual with those observed in delivery. In total, 36 h of the delivered intervention sessions will be coded (12 h of virtual interactive educational workshops, 12 one-to-one virtual outreach sessions and 12 multisite virtual facilitated meetings). These will be distributed across the delivery period, in accordance with the National Institutes for Health Behaviour Change Consortium recommendations. Eighty to 100% adherence to intervention specifications represents ‘high’ fidelity of delivery, 51 to 79% represents ‘moderate’ fidelity, and < 50% or less represents ‘low’ fidelity [31].

Our multi-stage analysis will occur concurrently with data collection to allow for emerging trends found in earlier fieldwork to be explored later. We will share interim analyses with stakeholders to identify additional avenues for exploration in later interviews and documentary analysis. This wider group will include clinicians, people with diabetes, policy leads and implementation scientists.

We will undertake workshops for integrative analysis of the different data sources (from interviews, observations and document analysis) to address the three investigative objectives of the process evaluation: implementation and engagement; fidelity and tailoring (1–2 half-day workshops per investigation). The analysis workshops will explicitly reconnect and explore the data and findings to develop higher level analyses with reference to NPT, organisational readiness and BCTs, and the matching of improvement strategies to barriers and facilitators using causal models [32]. Project team and stakeholders will be invited to these workshops as appropriate.

### Economic evaluation

To estimate value for money of NDA feedback with QIC, we will.

- Conduct a micro-costing of the quality improvement collaborative and local improvement strategies;
- Estimate the cost-effectiveness of NDA feedback with QIC versus feedback alone;
- Estimate the budget impact of NHS-wide QIC roll-out.

We will collect data through NDA data extraction, interviews, surveys and observations.

Micro-costing sampling and data collection: We will interview or survey the QIC delivery team and members of the intervention and control teams to map out the resources required to deliver the intervention. These are likely to span intervention refinement, delivery and response activities. We will create a record of consumable costs incurred (e.g. virtual delivery licence costs, printed material) and staff time (and grade) required, for intervention adaptation and delivery (NDA team). We will interview 15–20 intervention arm participants to understand costs associated with participation (e.g. NHS staff time for attending virtual sessions). We will cost additional activities that result from the intervention (e.g. meetings with stakeholders, local team training, additional consultations with patients). The interviews will take place after the virtual workshops and outreach sessions and at the end of intervention delivery. We will sample teams based upon initial performance. We will interview 8–12 control team members to assess costs associated with feedback alone.

### Micro-costing analysis

Staff time will be costed using national database unit costs [33] and combined total costs will be estimated for NDA feedback with QIC and for feedback alone. We will seek to capture the variance in costs that might occur across centres and incorporate this uncertainty in the analysis. We will also empirically estimate the denominator sample for deriving the per patient intervention cost.

### Cost-effectiveness design

This evaluation will be model-based and adopt the perspective of the health and social care provider. Analysis will be presented over a range of time horizons but, data permitting, a lifetime horizon will represent the base case.

### Cost-effectiveness data collection and sampling

The evaluation will adhere, as far as possible, to the NICE reference case [34]. We will not collect or analyse individual-level or centre-level data but will use existing published evidence and trial aggregate data to parameterise the model.

### Cost-effectiveness analysis

Economic evaluation outcomes are typically reported as incremental cost-effectiveness ratios (ICER) or net (monetary or health) benefit. Net (monetary) benefit is a rearrangement of the ICER and estimated as (λ x QALYs)–Costs, where λ is the willingness to pay threshold per health gain (in the case of NICE, £20,000-£30,000 per QALY). The primary analysis will present incremental net monetary benefit for NDA feedback with QIC versus feedback alone.

The value for money of insulin pumps has been evidenced by previous clinical and cost-effectiveness research, summarised in systematic reviews. This evidence was of sufficient weight to lead to a positive recommendation from NICE [2].

The current evaluation will not seek to re-estimate the value of the technology and will not therefore build a de novo economic model of type 1 diabetes. Instead, we will estimate the value of the alternative implementation strategies alone. As such, this evaluation uses the general principles of value of implementation [35]. A targeted review of relevant published literature, NICE appraisals and guidelines will identify trial and model-based economic evaluations of insulin pump cost-effectiveness in the UK context. Several of these are available [36, 37] as well as reviews in the area [38]. We will use selected studies to identify the most plausible estimates of net benefit along with (if appropriate) other candidate estimates to use in scenario analyses.

The value of the intervention will be defined as the most plausible incremental net benefit of insulin use (versus no use, i.e. multiple daily injections) multiplied by the probability of uptake, minus the costs of the improvement strategy for each arm. The probability of uptake will be derived from adjusted statistical estimates (e.g. as odd ratios). We will develop a simple decision tree model (DTM) to estimate cost-effectiveness which will use value for money (i.e. lifetime net benefit) as model pay-offs and incorporate probability of pump prescription and probability of sustained pump use. We will conduct extensive deterministic sensitivity analyses to test analytical assumptions and the values adopted. We will define distributions around analysis parameters (strategy cost, probability of uptake, net benefit of insulin use) and conduct a probabilistic sensitivity analysis. Some studies [39] report confidence intervals or variance around cost-effectiveness estimates which could be used for this purpose. Where these are not available, we will use existing studies to inform assumptions around likely distributions.

The analysis will report the incremental net benefit of NDA feedback with QIC versus feedback alone, and the probability that the QIC is cost-effective. We will explore the heterogeneity of value across key sub-groups (e.g. deprivation levels), thus providing distributional estimates of cost-effectiveness [40, 41].

We will conduct supplementary cost-effectiveness analyses using cost per change in blood glucose and cost per uptake in pump use as the estimates of effect. Our analysis plan will follow CHEERS reporting guidance [42]. A NICE willingness to pay threshold range of (£20,000–£30,000) per QALY will be assumed, discounting beyond year 1 at the NICE recommended rate (currently 3.5%).

### Budget impact data collection and sampling

We will use NDA data to estimate numbers of people with type I diabetes not currently using insulin pumps. We will use the costs estimated during micro-costing to determine the budget impact of intervention roll out across the NHS.

### Budget impact data analysis

Costs over year 1 and subsequent years (with likely time horizons including 2–5 years) will be estimated. Scenario analyses will test assumptions made and values incorporated in the analysis, e.g. around type 1 diabetes prevalence and incidence and intervention sustainability.

### Study progress

We have gained ethical and Health Research Authority approval. We have recruited and allocated 77 clusters, where a cluster includes up to 3 specialist teams within an organisation or patient pathway. This is the majority of diabetes specialist teams in England and Wales. More recent NDA data suggests that the control group may improve by 2%, rather than the estimated 3% figure used in our initial power calculations.

Although our actual recruitment figure is lower than planned (*n* = 120 teams), 77 clusters will allow us to detect an absolute increase of 8% in prescribing of insulin pumps (i.e. 2% in control arm versus 10% in intervention arm) whilst retaining 87% power. This assumes a parallel design without baseline measures. Therefore, after adjusting for baseline, the power should increase beyond what is estimated above. We therefore believe that our trial is sufficiently powered to detect a clinically important improvement. We have discussed this with our independent Project Steering Committee (Supplementary materials 1) who agree with this assessment.

## Discussion

Clinical performance improvement in response to national audit feedback may be limited by a lack of motivation to change [14, 16, 18] and the selection and enactment of improvement actions [19], including poor tailoring to the local context [20]. Theory [14] and policy [12, 43] recommend increasing the quality improvement capabilities of feedback recipients. We will test a theory-, evidence-, and stakeholder-informed intervention to enhance feedback recipients’ capabilities [19]. The intervention, a form of QIC, will be offered to all specialist diabetes teams in England and Wales. Teams will be allocated at random to intervention and waiting-list control arms. We will evaluate the impact upon the use of insulin pumps by these teams, as well as investigating the impact upon equity, patient outcomes, cost-effectiveness and exploring implementation and engagement with the QIC.

Our study illustrates how to advance scientific knowledge on how to reinforce the effects of audit and feedback through a rigorous evaluation embedded within a national audit programme. It responds to calls for empirically and theoretically informed research on interventions such as audit and feedback, particularly for more head-to-head trials of different ways of reinforcing feedback delivery [44].

We recognise the potential for contamination between sites. Whilst we anticipate this to be minimal, we will actively monitor and describe potential contamination sources (e.g. occurring through conferences and regional networking). Access to the intervention sessions will be restricted. During intervention sessions, we will ask participants to avoid actively sharing intervention experiences beyond the collaborative group during the evaluation period and detail the potential impact of contamination.

## Conclusion

Fifteen years after NICE guidelines recommended insulin pumps for people with type 1 diabetes and an HbA1c greater than 69 mmol/mol, many NICE-eligible people are not using insulin pumps. Gaps in the uptake of insulin pumps and high variations in use might relate to care delivery factors. The NDA QIC seeks to improve the quality improvement capabilities of national audit recipients as a route to addressing these care delivery factors and increasing the use of insulin pumps.

The specified QIC targets specific practices in the response to national audit data. Evaluating the delivery of the QIC will provide valuable insights to national audit providers and commissioners, diabetes policy-makers, practitioners and implementation scientists. If effective, the QIC will implement NICE-recommended care associated with improved patient outcomes.

### Supplementary Information

**Additional file 1.** EQUIPD Protocol further details.

## Data Availability

Data sharing is not applicable to this article as no datasets were generated or analysed during the current study. The intervention manual is available from the corresponding author (MS).

## Appendix

**Table 1:** TIDieR Framework [23] Intervention Description

Item number		
1	Brief name Provide the name or a phrase that describes the intervention	National Audit Quality Improvement Collaborative
2	Why: Describe any rationale, theory, or goal of the elements essential to the intervention	The development of commitment and informational appraisal to select actions, resonating with the theory of organisational readiness for change [17]
3	What materials: Describe any physical or informational materials used in the intervention, including those provided to participants or used in intervention delivery or in training of intervention providers	The workshop includes slides to increase the coherence and cognitive participation of the target behaviours described in the logic model. These were supported by online materials to support participants to identify influences upon participation using the Theoretical Domains Framework, align these influences to actions and to identify stakeholder influence and interest
4	What procedures: Describe each of the procedures, activities, and/or processes used in the intervention, including any enabling or support activities	The intervention is described in a manual The active ingredients are described in the logic model
5	Who provided: For each category of intervention provider (e.g. psychologist, nursing assistant), describe their expertise, background and any specific training given	National Diabetes Audit Quality Improvement Lead, the DiabetesUK Engagement Lead, the National Diabetes Audit Clinical Lead and clinicians with expertise in improvement and insulin pump use
6	How: Describe the modes of delivery (e.g. face-to-face or by some other mechanism, such as internet or telephone) of the intervention and whether it was provided individually or in a group	Virtual delivery through MS teams and using Google JamBoard
7	Where: Describe the type(s) of location(s) where the intervention occurred, including any necessary infrastructure or relevant features	Virtual delivery through MS teams and using Google JamBoard
8	When and how much: Describe the number of times the intervention was delivered and over what period of time including the number of sessions, their schedule, and their duration, intensity or dose	Two virtual workshops (6 h in total), two virtual outreach sessions (30 min in total) and 12 facilitated, virtual meetings (1 h each) Participants are expected to attend eight facilitated, virtual meetings
9	Tailoring: If the intervention was planned to be personalised, titrated or adapted, then describe what, why, when, and how	Not applicable Note: Tailoring work is undertaken by intervention participants

## Abbreviations

BCT: Behaviour change techniques
CTRU: Clinical Trials Research Unit
EQUIPD: Evaluation of quality improvement for people with diabetes
ERIC: Expert Recommendations for Implementing Change
HbA1c: Haemoglobin A1c (a measure of glucose control)
HQIP: Health Quality Improvement Partnership
ICC: Intra-cluster correlation co-efficient
ICER: Incremental cost-effectiveness ratios
ISRCTN: International Standard Randomised Controlled Trial Number
ITT: Intention to treat
λ: Willingness to pay threshold per health gain
NDA: National Diabetes Audit
NICE: National Institute for Health and Care Excellence
NPT: Normalisation Process Theory
PMG: Project Management Group
PSC: Project Steering Committee
QALY: Quality adjusted life years
QIC: Quality Improvement Collaborative
TDF: Theoretical domains framework
TIDieR: Template for Intervention Description and Replication
