# Supplementary material for: A cluster randomised controlled trial, process and economic evaluation of quality improvement collaboratives aligned to a national audit to improve the care for people with diabetes (EQUIPD): study protocol
Source: Implement Sci. 2023 Aug 31;18:37. doi: 10.1186/s13012-023-01293-0 (PMC10470130; doi:10.1186/s13012-023-01293-0)
Supplement: Supplementary file 1 — Additional file 1. EQUIPD Protocol further details. [file 13012_2023_1293_MOESM1_ESM.docx]

**Supplementary materials 1**

**Sample size calculation**

The proposed sample size provides 90% power to detect a 7% increase in the proportion of those patients moving onto a pump for at least three months (3% in the control arm and 10% in the intervention arm) at a 5% significance level after adjustment for clustering and loss to follow-up.

Determination of clinical significance accounted for comparisons and ranges of effects from the Cochrane review of audit and feedback [9], advice from Experts by Experience, funder expectations and clinical perspectives about maximum feasible improvement. We considered it plausible and feasible that specialist diabetes teams could initiate an insulin pump and provide support for an average of one extra person a week. We assumed an intra-class correlation co-efficient (ICC) of 0.14 to account for clustering by specialist team, a coefficient of variation of 0.72 to account for variation in the number of patients per team and 10% loss to follow-up. Estimates for the clustering effects, cluster size and control arm proportion are based on NDA data from 2019-20.

**Recruitment and withdrawal procedures**

The invitation from the NDA to take part in the Quality Improvement Collaborative (QIC) will be directed to the clinical leads at each site and will describe the aim, content, delivery, random allocation and timing of the QIC. The invitation will ask them to provide the names, roles and emails of two people who will form the sites’ QIC team. During the initial virtual meeting with the team lead, discussion will identify a potential third member of the QIC team.

Individual patient consent will not be required. No additional patient data will be collected beyond what is already collected as part of the NDA. If patients do not wish to have their data recorded in the NDA, patients can withdraw using the National Data Opt-Out electronic form at any time. This will result in no further data being collected. The national opt-out does not remove previously collected data.

Teams can withdraw from participating in the intervention at any time without having to give a reason. If a team withdraws consent to participate, clarification will be sought on whether withdrawal is from the intervention, or from the evaluation. Teams which wish to withdraw from the intervention remain required to submit their data to the NDA. Teams which have agreed to take part in the evaluation will not be able to withdraw their NDA data from the analysis.

**Standard feedback provided to both intervention and control arms**

Standard feedback comprises a publicly available annual national report and dashboard describing pump use as a proportion of caseload, identifying the selected sites and other sites across England. It also describes performance in pump and non-pump users in terms of care process completion and treatment target achievement for the site and all sites. The national report describes key recommendations, methodology, participation and data quality information, differences in pump use by ethnicity, age, sex and deprivation and a national-level description of the data described at site-level.

**Data extraction**

Insulin vial prescription data and HbA1c measurements are routinely extracted from GP clinical systems via the General Practice Extraction Service; the practice must approve the extraction. Specialist diabetes services submit caseload data and additional HbA1c and pump use data through the Clinical Audit Platform. People will be identified as receiving care from a specialist service if they are included in the caseload data provided through the Clinical Audit Platform or where linkage with Hospital Episode Statistics indicates that they attended diabetes/endocrinology outpatient appointments in the relevant time period.

**Study management**

The Project Management Group (PMG) is made up of the study team and will meet at key points during the study to oversee the study including the set-up, on-going management, promotion of the study and the results. The Project Steering Committee (PSC) will provide overall supervision of the programme, in particular progress, adherence to protocols, safety and consideration of new information. It will include an Independent Chair and five other independent members, including patient representation. The Chief Investigator and other members of the PMG may attend the PSC meetings and present and report progress. The Committee will meet annually as a minimum.
